# Impact of Biological and Lifestyle Factors on Cognitive Aging and Work Ability in the Dortmund Vital Study: Protocol of an Interdisciplinary, Cross-sectional, and Longitudinal Study

**DOI:** 10.2196/32352

**Published:** 2022-03-14

**Authors:** Patrick D Gajewski, Stephan Getzmann, Peter Bröde, Michael Burke, Cristina Cadenas, Silvia Capellino, Maren Claus, Erhan Genç, Klaus Golka, Jan G Hengstler, Thomas Kleinsorge, Rosemarie Marchan, Michael A Nitsche, Jörg Reinders, Christoph van Thriel, Carsten Watzl, Edmund Wascher

**Affiliations:** 1 Leibniz Research Centre for Working Environment and Human Factors (IfADo) at the Technical University of Dortmund Dortmund Germany

**Keywords:** cognitive aging, genetic polymorphisms, immunology, metabolism, latent infections, stress, occupational health, cardiovascular system, neuropsychology, magnetic resonance imaging, electroencephalography, lifespan, lifestyle, longitudinal study, biomarkers, longevity, aging.

## Abstract

**Background:**

Previous research revealed several biological and environmental factors modulating cognitive functioning over a human’s lifespan. However, the relationships and interactions between biological factors (eg, genetic polymorphisms, immunological parameters, metabolic products, or infectious diseases) and environmental factors (eg, lifestyle, physical activity, nutrition, and work type or stress at work) as well as their impact on cognitive functions across the lifespan are still poorly understood with respect to their complexity.

**Objective:**

The goal of the Dortmund Vital Study is to validate previous hypotheses as well as generate and validate new hypotheses about the relationships among aging, working conditions, genetic makeup, stress, metabolic functions, the cardiovascular system, the immune system, and mental performance over the human lifespan with a focus on healthy working adults. The Dortmund Vital Study is a multidisciplinary study involving the Departments of Ergonomics, Immunology, Psychology and Neurosciences, and Toxicology at the Leibniz Research Centre for Working Environment and Human Factors at the Technical University of Dortmund (IfADo) in Germany, as well as several national and international partners.

**Methods:**

The Dortmund Vital Study is designed as a combined cross-sectional and longitudinal study. Approximately 600 healthy subjects aged between 20 and 70 years will participate. A wide range of demographic, psychological, behavioral, sensory, cardiovascular, immunological, and biochemical data, a comprehensive electroencephalography (EEG)-based cognitive test battery as well as structural and functional magnetic resonance imaging (MRI) have been included in the study.

**Results:**

The study was approved by the Ethics Committee of IfADo in October 2015. The baseline testing was conducted between 2016 and 2021 and will be repeated every 5 years (3 follow-up measures until 2035). As of March 2020 (until the outbreak of the COVID-19 pandemic), 593 participants have been enrolled. Some results from the cross-sectional part of the study were already published, further results will be published soon. Longitudinal data will be analyzed and published by 2025.

**Conclusions:**

We anticipate that the study will shed light on sources of interindividual differences in the alterations of cognitive functioning with increasing age and reveal biological and lifestyle markers contributing to work ability, longevity, and healthy aging on the one hand, and to risk factors for cognitive decline, mild cognitive impairment, or even dementia on the other hand.

**Trial Registration:**

ClinicalTrials.gov NCT05155397; https://clinicaltrials.gov/ct2/show/NCT05155397

**International Registered Report Identifier (IRRID):**

DERR1-10.2196/32352

## Introduction

### Background

Cognitive performance in humans is a complex phenomenon that is influenced by numerous variables. Among them are age [1,2], various infections [3,4], inflammatory processes often due to the release of cytokines from immune cells [5,6], metabolic alterations such as hyperammonemia [7,8], lifestyle factors including physical and mental activity, type of work, nutrition and stress [9-16], as well as numerous genetic variants [17,18] that can be analyzed by polygenic scores [19]. Most studies in this field of research have focused on specific individual factors of influence and did not consider the development of individuals over a significant part of their lifetime. To bridge this gap, we initiated the Dortmund Vital Study that assesses cognitive performance in a longitudinal study design using a complex multiscale approach to understand the influence of the most important lifestyle, occupational, and biological factors on cognitive performance.

### Research Questions and Aims

The aim of the Dortmund Vital Study is to evaluate the effect of several endogenous and exogenous parameters and their complex interaction on behavior and the underlying neuronal activity. This will be achieved by implementing cross-sectional and longitudinal designs. By repeated measurements of the endogenous and exogenous parameters over an extended period, risk factors for possible cognitive impairment associated with depression, occupational burnout, or age-related diseases, such as mild cognitive impairment or dementia, and nonpathological cognitive decline shall be identified. More importantly, the Dortmund Vital Study focuses on the following research aspects, which are currently poorly understood and largely unexplored:

1. Liver Immune Behavior-Brain Axis: A core question is how specific aspects of cognitive performance and brain activities as well as the brain structure are affected by metabolic products, inflammatory mediators, or viral infections, including concentrations of metabolites such as creatinine, ammonia, or immunological parameters in the blood such as inflammatory cytokines, and novel metrics of immunological age. Combinations of standardized cognitive tests and electroencephalographic techniques with computer-based tests, as well as structural and functional magnetic resonance imaging (MRI) will be used to explore cognitive performance with a much greater level of detail compared to previous studies.
2. Multidimensional and Multichannel (Omics) Analyses: The roles of metabolic parameters or inflammation mediators have traditionally been determined by analyzing individual effects of the selected parameters per study. However, with currently available techniques and technologies, it is possible to measure a wide range of parameters (ie, metabolites, proteins, RNA species, DNA variants, and immune cells) simultaneously, using the so-called “omics” techniques or multichannel analysis. Consequently, a more comprehensive picture is obtained, which can then be evaluated using systems biology techniques to understand the interactions among the individual factors.
3. Age-Related Perspective: Temporal dynamics of the interplay between endogenous versus exogenous factors and cognitive functions, as well as work ability over the lifespan will be assessed. In particular, the effect of metabolic products, infections or inflammatory processes, and the brain structure and function on neurobehavioral parameters in young, middle, and old age will be analyzed in a cross-sectional design.
4. Disease-Related Perspective: With the present design, the derivation and evaluation of risk factors for widespread mental and neurodegenerative diseases such as depression, burnout, mild cognitive impairment, or dementia will be addressed. Moreover, epigenetic approaches that investigate the influence of environmental changes on the genome, as well as on the brain structure and function will be feasible. Epigenetic dysregulation is also important for the development of immunological and neuronal diseases and complex developmental disorders.
5. Genetic Perspective: Although it is known that genes influence cognitive performance, which in turn is associated with the structural and functional network efficiency of the brain, functional understanding of the specific brain parameters that mediate the relationship between individual genetic variations or polygenic scores and cognitive performance is incomplete. Given that the current study will capture all relevant neurocognitive measures, it will be possible to investigate the interactions among genes, brain parameters, and cognitive performance.
6. Longitudinal Perspective: Most existing studies have only used cross-sectional study designs that limit the interpretation of results, given that the results obtained in cross-sectional studies do not imply causality. Therefore, this study has been additionally designed as a longitudinal study with postmeasures every 5 years. The advantage of such a longitudinal study is that parameters, namely particular metabolic or immunological states, genetic variations, or brain metrics, can be recorded at an early stage and then be tracked to test whether they result in any effect later; moreover, one can investigate how the state of these parameters changes over the course of a person's life. This design allows causal inferences.

The benefit of the Dortmund Vital Study will be that profound knowledge regarding the interactions among metabolism, the immune system, genetics, the brain structure and function, and cognition will be gained. In the long run, this knowledge will lead to empirically based intervention strategies to preserve cognitive functioning, work ability, and promote healthy cognitive aging. The strength of the study design lies in the fact that variables with an impact on cognitive performance and their interactions can be determined comprehensively and that factors whose significance is still unknown today will be identified and evaluated. Due to the complex design and several measurement parameters, a wide range of research questions is possible. Given below are 4 of several hypotheses possible in the study.

#### Hypothesis 1: Background and Mechanisms of Stress-Related Exhaustion Disorder

Burnout, a synonym for stress-related exhaustion disorder is a widespread phenomenon, but the underlying mechanisms have not yet been sufficiently explored. In addition, there is no clear distinction between burnout and depression [20,21], and there is currently limited evidence connecting burnout and cognitive functions [22,23]. However, it is known that the reward system in the orbitofrontal brain areas is altered owing to depression and subclinical depressive symptoms, which is evident in electrophysiological correlates [24]. As a result, the perception of mistakes or negative feedback is increased whereas the sensitivity to rewards is decreased [25]. The reward system is based on complex cognitive abilities, the so-called executive functions, which enable goal-directed behavior. Therefore, it is important to compare the effects of burnout and depression using the same parameters and measures and identify similarities and differences with respect to executive functions between the 2 disorders. We expect that individuals with high scores on the burnout scales will perform poorly in cognitive tasks and in tests measuring executive functions, such as working memory, task switching, decision-making, or distractibility. Additionally, we aim to explore possible differences in the resting-state electroencephalography (EEG) and functional MRI brain activity between burnout and nonburnout employees; we expect reduced alpha power and cortical hyperactivity in the former group [26] that may be the neuronal correlate of hypersensitivity to negative events. However, no effects are expected in tasks that measure basal cognitive functions such as vigilance or processing speed. Moreover, a relationship between burnout and immunological parameters [27,28] or metrics of immunological age can be assumed. For example, the concentration of hair cortisol as an index of long-term stress is expected to be positively related to the severity of burnout symptoms and reduced cognitive performance [29,30].

#### Hypothesis 2: Influences of Biological Factors on Cognitive Functioning Over the Lifespan

Considerable progress has been made in recent years to identify biological factors that influence cognition in older adults. These include genetic polymorphisms, such as the brain-derived neurotrophic factor (BDNF) Val^66^Met [31], catechol-O-methyltransferase (COMT) Val^158^Met [32], or latent infectious diseases (eg, *Toxoplasma gondii*) [33,34] that affect brain metabolism. However, studies with young individuals often show no effects [3]. Our hypothesis is that the effects of genes or neuropathologically relevant infections increase with age, which is probably due to the decreasing integrity of neuronal networks as one ages. To test this hypothesis, we aim to compare the performance of young, middle-aged, and old participants with and without infections, such as *Toxoplasma gondii* or COVID-19, or those with a specific gene expression pattern, with the expectation that there are larger inter- and intraindividual differences in the cognitive parameters in older adults compared to younger adults. Moreover, as this is a long-term study, we have the opportunity to test these hypotheses of altered performance in the same individuals over many years. We also aim to identify the immunological age of the participants based on various immunological parameters [35], which will allow us to determine if immunological age rather than chronological age is associated with changes in cognitive functioning.

#### Hypothesis 3: Interaction of Damaging and Protective Factors

Cognitive age is influenced by several internal and external factors. Apart from genetic makeup, environmental and lifestyle variables such as education, type of work, habitual physical and mental activity, nutrition, and stress at work play a modulating role and explain the high degree of variability observed with cognitive performance at advanced ages [36,37]. Thus, we aim to determine which of these factors critically influence the development of cognitive competence and the so-called cognitive reserve in older adults [38]. Consequently, we can discern whether, for example, long-term stress neutralizes the benefits of advanced education on cognitive aging, or whether regular physical activity can compensate for the negative effects of less advanced education. On the other hand, we expect that psychosocial stress (whether during childhood or adulthood) influences immunological or metabolic parameters [39] and that individuals suffering from permanent stress, which has been demonstrated for particular occupational groups, will show corresponding changes in their metabolism and cognition [40].

#### Hypothesis 4: Analysis of Factors Affecting Work Ability Across the Lifespan

As employees age, their physical and mental abilities tend to decline and the risks of accidents at work and work-related diseases increase [41,42]. Work ability is defined as the relationship between individual resources and specific work requirements, and it is the result of interactions between job requirements in terms of physical and mental strain, capacities and skills of the employees, as well as their health status and subjective evaluation of functioning in a given working environment. The instrument used to evaluate the ability to work is the Work Ability Index (WAI) [43]. It considers specific psychosocial and physical factors related to performing a given type of work, as well as the employee’s mental and physical resources. Therefore, it is important to understand the relationships among age, lifestyle, quality of life, work-related factors, stress-related impairments, and cognitive abilities, as well as examine work ability measured by the WAI. Additionally, we will consider individual health risks, such as cardiovascular, metabolic, anthropometric, or immunological contributors to work ability. The longitudinal study design helps assess changes in several risk factors associated with compromised work ability to promote healthy aging in working environments and reduce the likelihood of early retirement.

## Methods

### Study Population

The participants of the Dortmund Vital Study (trial registration number: NCT05155397) represent a sample of a generally healthy population of a western society aged between 20 and 70 years. We define “healthy” in a rather broad sense and allow the inclusion of individuals who are smokers, drink alcohol, are overweight, or have a history of diseases without having severe symptoms. Moreover, we do not impose restrictions regarding education or occupation to enhance the representativity of the sample. Participants are recruited via an internet site, newspaper advertisements, reports and announcements in local print and radio media, public information events, social media, and flyers throughout the city of Dortmund, Germany. In addition, some larger companies in the region were contacted and asked to inform their employees about participating in the study. The number of subjects resulted from a biostatistical estimation (power analysis), in which the size of the expected effects and their variance were considered. The study parameters will be collected repeatedly for 4 time points, namely at the start of the study (baseline), and then 5, 10, and 15 years later (time points for measuring within-subject factors) and between 3 age groups (between-subject factor ie, Age Group: young, middle-aged, and old). Of particular interest here is the interaction between the age group and the measurement time point, which will provide insight into the different trajectories within the specific age groups. Thus, power estimation was conducted exemplarily for repeated-measures analyses of variance (ANOVA) of within-between interactions. The sample size was determined based on a small effect size of *f*=0.1 for the mixed ANOVA model with repeated measures (4 time points) and interactions with the Age Group factor (3 categories), given an error probability of α=.05 and a power of 0.95, resulting in a sample size of 264 individuals (determined with G*Power, a free-source statistical software package) [44]. However, having a larger number of subjects is desirable owing to expected dropouts over the long duration of the study. Therefore, a group size of approximately 600 was targeted, as we assumed a dropout rate of up to 20% per time point of the follow-up measures. Assuming this dropout rate, the number of remaining participants 20 years later would be approximately 300, which roughly corresponds to the estimated sample size, as summarized in Table 1.

**Table 1:** Longitudinal study design showing birth cohorts of the study from 1946 to 1996 with measurements at 5-year intervals^a^.

Birth cohort	Age at T1 2016-2021	Age at T2 2021-2026	Age at T3 2026-2031	Age at T4 2031-2036
1996	20 years	25 years	30 years	35 years
1946	70 years	75 years	80 years	85 years
n	600	480	384	307

^a^The ages of the youngest (20 years old at baseline) and oldest (70 years old at baseline) cohorts and the expected number of subjects at 4 test points (T1 to T4) are indicated.

### Representativeness of the Sample

To verify the representativeness of the sample and thus the generalizability of the future findings, four different aspects are considered:

1. Age Distribution and Gender: We enroll almost the same number of participants in each age group and compare the proportion in each decade of life (as well as the proportion of women and men) between the participants in this study with the corresponding proportion of the general population in Germany.
2. Genetic Representativeness and Homogeneity: We test whether the distribution of genetic polymorphisms in the sample does not differ from the Hardy-Weinberg equilibrium (HWE).
3. Cognitive Abilities: We test whether neuropsychological tests measuring crucial cognitive functions are comparable with reference values in the literature.
4. Education and Occupation: We compare levels of education and the proportion of employed individuals in the present sample and in the general population in Germany.

First, until August 2021, totally 593 participants have been enrolled. Compared with the average of 20- to 70-year-old people living in Germany [45], the participants of the Dortmund Vital Study are slightly younger (44.2 vs 45.8 years on average), and the proportion of women is higher (61.5% vs 49.6%). The proportions of participants in the Dortmund Vital Study in 5 age groups (20 to 29 years: n = 122, 20.6%; 30 to 39 years: n = 113, 19.1%; 40 to 49 years: n =110, 18.5%; 50 to 59 years: n =146, 24.6%; 60 to 70 years: n =102, 17.2) correspond largely to the proportions in the German population (20 to 29 years: 17.2 %; 30 to 39 years: 19.6 %; 40 to 49 years: 18.2 %; 50 to 59 years: 24%; 60 to 70 years: 21%).

Second, to ensure that the sample of participants of the Dortmund Vital Study is representative for the population in a genetic manner, the HWE is used to compare the expected and observed proportion of common homozygote, heterozygote, and rare homozygote variants of the single nucleotide polymorphisms (SNPs) calculated by the internet-based HWE calculator [46]. A deviation from the HWE was found in only 1 out of the 19 measured SNPs (IL-12), as shown in Table 2.

Third, we compared the scores in the neuropsychological tests between the present study and the corresponding reference or normative values available in the test manuals or literature. The Mini Mental State Examination (MMSE) used for detecting early signs of cognitive impairment shows a mean of 28.1 points (SD 1.9) out of a possible 30 points in our participants aged 60 years and older (n=102) and 28.3 points (SD 1.6) in a healthy sample of 204 participants in the same age range in the study by Votruba et al [47]. For the Beck Depression Inventory, the mean score in our sample is 5.7 points (SD 6.4), which is well below the cutoff for minimal depression (9 points), and similar to the mean of a healthy control group (n=583) described in the test manual, which scored 7.4 points (SD 7.3) [48]. The Multiple-Choice Vocabulary Test (MWT-B) that measures premorbid (crystalized) intelligence shows a mean score of 31 points (SD 3.2) that corresponds to a mean IQ of 115. A healthy control group of adults (n=102) in a study by Satzger et al [49] reached a mean IQ of 121 (the test usually overestimates the IQ obtained with typical intelligence tests). For the digit span test from the Hamburg-Wechsler-Intelligenztest für Erwachsene, Revision (HAWIE-R), the German version of the Wechsler Adult Intelligence Scale-III [50], the mean number of correct responses is 14.1 (SD 3.6) in the present sample and 15.0 (SD 3.2) in a healthy adult sample (n=100) in the HAWIE-R manual [51]. Similarly, the Digit Symbol Test from the same test battery shows a mean score of 59.1 points (SD 12.3) for the Dortmund Vital Study participants and 50.6 (SD 10.3) points in the HAWIE-R manual. For the Stroop Test from the Nürnberger Alters Inventar (NAI) [52], the time to complete the word list for participants aged between 55 to 69 years is 14.7 (SD 2.3) seconds in the present study (n=144) and 16.0 (SD 2.0) seconds in the reference values for healthy older persons in the manual (n=78). For the color naming list of the Stroop Test, the corresponding times are 21.1 (SD 3.7) versus 24.0 (SD 7.0) seconds, and for the interference list, they are 36.7 (SD 7.4) versus 44.0 (SD 13.0) seconds. The capacity for learning new words assessed by the Verbal Learning and Memory Test (VLMT) [53], the German version of the Rey Auditory Verbal Learning Test (RAVLT) [54], shows a mean sum of 54.8 points (SD 4.3), indicating the number of successfully learned words in our sample (n=525), and exactly 54.8 points in a norm sample (n=515) provided in the test manual. Furthermore, the D2 test, measuring selective attention and attentional endurance (D2-R) [55] reveals for our participants aged between 20 and 60 years (n=489), the mean number of correctly crossed symbols is 158 (SD 36.7), whereas the normative value for the same age group (n=976) in the manual is 153 (SD 28.4). Finally, for the 2 subtests from the Leistungs-Prüf-System (LPS, meaning performance test system) validated for people older than 50 years [56], our participants aged 50 years and older (n=217) had a mean of 24.7 (SD 5.1) correct responses in the logical reasoning and 19.5 (SD 6.4) in the spatial rotation subtests, whereas the controls in the LPS manual reached 22.0 (SD 5.2) and 17.6 (SD 6.0) correct responses, respectively.

Fourth, with respect to education, 28% (n=161) of the participants of the Dortmund Vital Study have secondary degree, 30.3% (n=174) have a high school diploma and 41.6% (n= 239) have a university degree, compared to the corresponding proportions in the German population (23.5%, 33.5%, and 18.5%, respectively). Furthermore, 68.6% of the participants in the Dortmund Vital Study are employed (full-, half-, or part-time) compared to 67.7% of the general population in Germany [45].

In summary, we consider the sample of the present study as representative in terms of age distribution, genetics, depressive symptoms, cognitive parameters, and occupation. However, in contrast to the general population, the ratio of female participants and that of university degree holders appear to be higher than in the general population.

### Inclusion Criteria

The study includes adults without history of severe diseases, namely neurological diseases such as dementia, Parkinson disease, or stroke; cardiovascular diseases; bleeding tendency; oncological diseases; psychiatric disorders such as schizophrenia, obsessive-compulsive disorder, anxiety disorders, or severe depression; head injuries; head surgery; head implants; eye diseases (cataract, glaucoma, or blindness); accidents that limit physical fitness and mobility; and those who do not use psychotropic drugs and neuroleptics. Medications that did not lead to exclusion from the study are blood thinners, hormones, antihypertensives, and cholesterol reducers. Participants have normal or corrected-to-normal vision and hearing and fulfill standard inclusion criteria for MRI measurements.

### Telephone Interview

Once the participants register via a contact form, they receive a telephone call where an interview is conducted to check their inclusion criteria and obtain personal data, such as age, contact information, education, current occupation (type, full time/part-time), planned changes in the next years (relocation, retirement), pre-existing and current illnesses, physical restrictions, medication, willingness to visit the Leibniz Research Centre for Working Environment and Human Factors at the Technical University of Dortmund (IfADo) for tests on 2 independent days, and the willingness to participate in follow-up tests in 5, 10, and 15 years.

### Measures

**Figure 1:**
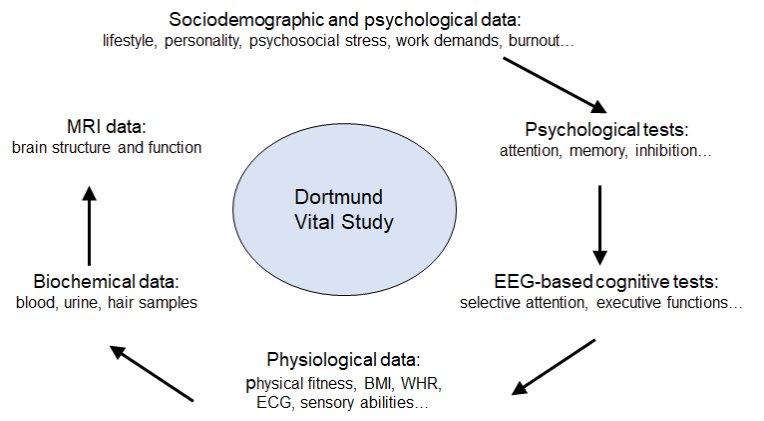
Schematic illustration of the measures used in the Dortmund Vital Study. The arrows indicate the order in which the data are collected. Beginning with the first post-test, an MRI will be included into the test battery. EEG: electroencephalography, ECG: electrocardiography, MRI: magnetic resonance imaging.

The Dortmund Vital Study uses a wide range of instruments to measure biological and environmental parameters that potentially affect cognitive performance across the lifespan, as shown in Figure 1.

#### Questionnaires

The participants receive several questionnaires via mail that should be filled in at home and brought to the first test session. The battery includes nonstandardized and standardized questionnaires. Nonstandardized questionnaires obtain sociodemographic and lifestyle variables (marital status, children, education, proficiency in languages other than the native language, type and history of employment, history of physical activity, nutrition, smoking, social activities, hobbies, using digital media, caregiving of family members), as well as request information on vision and use of glasses, necessary medical information for blood collection, and a rating of subjective time perception. Moreover, for the follow-up measurements starting in the middle of 2021, a questionnaire addressing COVID-19–specific aspects has been included that gathers information on, for example, the participants’ experience with the pandemic, how they coped with the pandemic, and the consequences of a COVID-19 infection (if applicable). Standardized questionnaires are used to measure the following: depressive symptoms (Becks Depression Inventory) [57], personality traits (Big Five Personality traits, NEO Five-Factor Inventory) [58], traumatic experiences during childhood with the adapted version of the Childhood Trauma Questionnaire [59], chronotype (D-MEQ) [60], cognitive failures in daily life (Cognitive Failure Questionnaire) [61], Grit personality trait (Grit Scale) [62], handedness (Edinburgh Handedness Inventory) [63], emotional dissonance [64], job control [65], physical activity (Lüdenscheid Physical Activity Questionnaire) [66], burnout (Maslach Burnout Inventory-General Survey [67], and Oldenburg Burnout Inventory [68], stress reactivity (Perceived Stress Reactivity Scale) [69], affectivity (Positive and Negative Affect Schedule) [70], psychosocial stress (Psychosocial Stress Questionnaire) [71], general self-control and self-control at work [72], chronic stress (Trier Inventory of Chronic Stress) [73], quality of life using the short version of the World Health Organization Quality of Life questionnaire (WHOQoL-BREF) [74], and work ability (WAI) [43,75]. For the follow-up tests, the Copenhagen Psychosocial Questionnaire-III [76] will be included to assess psychosocial work demands. Some of the questionnaires were adapted to suit the purpose of the present study.

#### Sensory Testing

To evaluate basic sensory abilities, audiometry and visual acuity tests are conducted. Audiometric thresholds are tested for 10 pure-tone frequencies (125, 250, 500, 750, 1000, 2000, 3000, 4000, 6000, and 8000 Hz) for the left and right ears (Oscilla USB100, Inmedico).

Visual acuity is measured using Vistec's Optovist according to the DIN 58220-3 standard “Visual acuity testing - Part 3: Test for use in expertise” for far vision with the right and left eye separately (monocular) and with both eyes together (binocular), and if available with correction for distance (glasses for far vision). The “inclined optometer” [77] is used to determine the zones of sufficient binocular vision at horizontal gaze inclination. For this, the near and far points are obtained, if available with distance correction (glasses for far vision). Moreover, the participants complete a questionnaire regarding the glasses they wear, and the visual and musculoskeletal complaints during the use of computer monitors at the workplace.

#### Neuropsychological Assessment

A wide range of cognitive functions are evaluated using the following standardized neuropsychological tests: evaluate the memory span and working memory (WM) using digit span forward and backward from HAWIE-R [51], the German version of WAIS-IIIR [50]; semantic memory in written and spoken versions (Word-Fluency Test from LPS) [56]; selective attention and attentional endurance (D2-R) [55]; lateralization and motor functions (Perdue Pegboard Test) [78]; crystallized intelligence (MWT-B) [79]; general cognitive status (MMSE) [80], different aspects of verbal memory like learning performance and retrieval (VLMT) [53], a German version of the RAVLT [54], psychomotor performance and speed of processing (Digit Symbol Test from HAWIE) [51], interference control and inhibition (Stroop Test) [81] from NAI [52], task switching (Trail Making Test, TMT-A and TMT-B) [82], and 2 subtests from the performance testing system [56] measuring logical reasoning and spatial rotation (refer to [83] for details of the tests). For the follow-up measurements, Raven’s 2 [84] will be included to estimate fluid intelligence and the number connection test [85] for measuring processing speed. Finally, all cognitive measures will be used to generate the *g*-factor also known as general intelligence. The *g*-factor is a construct developed in psychometric studies of cognitive abilities and human intelligence. It is based on the observation that performance of different cognitive tasks is positively correlated, reflecting the fact that an individual's performance of 1 type of cognitive task tends to be comparable to that person's performance of other cognitive tasks [86].

#### Computer-Based Cognitive Tests With EEG Recording

Behavior and electrical brain activity are simultaneously measured using 2 separate test batteries consisting of a total of 11 computer-based cognitive tasks measuring crucial cognitive functions. The test batteries are applied on 2 examination days. Before the start of the test batteries on days 1 and 2, EEG activity is measured for 2 minutes with eyes open, and 2 minutes with eyes closed to evaluate the resting-state oscillatory brain activity. To assess potential time-on-task effects on brain activity, these EEG measurements are repeated posttesting after the cognitive tests are completed.

The computerized test battery on day 1 includes the following tasks:

##### Bar Task [87]

The aim of this task is to evaluate attentional performance in a perceptual control task. The task is to respond to luminance changes in 1 of 2 symmetrically presented bars and to press the corresponding response key.

##### Psychomotor Vigilance Test [88]

The standard 10-minute psychomotor vigilance test measures sustained or vigilant attention by recording response times to visual stimuli that occur at random interstimulus intervals. The 4 interstimulus intervals are 2, 3, 5, and 8 seconds.

##### Simon Task [89,90]

The Simon task measures stimulus-response compatibility and conflict processing. The Simon effect elucidated in the task refers to the observation that spatially arranged responses to nonspatial stimulus features (eg, shape, color) are faster when the task-irrelevant stimulus location and the response are on the same side than when they are on opposite sides.

##### AX-Continuous Performance Task (AX-CPT) [91]

The AX-CPT is used to measure updating and strategy learning. It has been commonly used to examine shifts in the use of proactive and reactive cognitive control. The AX-CPT requires participants to respond to a certain cue-probe pair (ie, target cue-target probe; AX trials) and to withhold their response or make an alternate response or use other cue-probe pairs. The proactive control mode has been associated with cue-driven processing. In contrast, the reactive control mode has been associated with probe-driven processing. The shift between these alternative control modes can be assessed by comparing different cue-probe combinations.

##### Speech-in-Noise Perception Task [92]

This task measures speech understanding and auditory distractibility. This is examined in a simulated auditory stock market scenario in which the subjects must respond to a target company and its market value. This target company is included in 50% of the trials, the price of which is 50% above or below the critical value of 5. The target company and value are presented in the presence of 2 competing companies to provide distractive stimuli that must be inhibited.

The test battery on the second testing day primarily assesses executive functions.

##### n-Back Task [83,93]

The *n*-back task is assumed to be a measure of WM capacity because it requires maintaining, continuous updating, and processing of information. The task consists of a 2-choice condition with low WM-load (0-back) and a 2-back condition with high WM-load. Participants are successively presented with a series of visual stimuli; for each stimulus, they are asked whether it matches a stimulus presented *n* trials before. For example, in a *2*-back task, in which the stimuli consist of letters, participants must decide whether the current letter is the same as the letter in trial *n*–2.

##### Task Switching (Cue- and Memory-Based Task Switching) [94,95]

This task switching paradigm is frequently used to evaluate several control processes by applying only 1 experimental task consisting of different experimental blocks. It enables assessment of task preparation, WM, interference processing, and switching processes, as well as their interaction. First, participants are asked to perform 3 different single task conditions using the same stimulus material (numerical, parity, and font size tasks) in separate blocks of trials. In the next step, they are asked to switch between the 3 tasks in a randomized order using a cue stimulus that signals the relevant task in advance. In the last block, they switch between the tasks every third trial without any cue stimuli, requiring memorizing and recalling the task sequence.

##### Auditory Distraction [96,97]

This auditory task evaluates the ability to focus on a given task and ignore concurrent distracting stimuli. Participants perform a duration discrimination task on a random sequence of long- and short-tone stimuli, which either have a standard pitch (80%) or a deviant pitch (20%). EEG correlates of involuntary shifts in attention to the task-irrelevant deviant stimuli and subsequent reorientation to the task-relevant stimulus feature are evaluated.

##### Interference Processing [81,98]

The Stroop task measures the susceptibility to interference and the capacity to inhibit irrelevant stimuli and prepotent responses. Participants must indicate the meaning or the color of color words whereas the word color is either congruent or incongruent with the word’s meaning. One-half of the trials are congruent (name and color are the same), the other half are incongruent, and both types of trials are presented in a randomized order. As word reading is an automated response and produces no or little interference in congruent trials, naming of the font color and inhibiting the word’s meaning in an incongruent trial is a complex executive function that produces strong interference.

##### Cognitive Inhibition (Go/NoGo) [99,100]

A standard task to evaluate inhibitory control is the Go/NoGo task, in which participants are asked to respond under time pressure to frequent stimuli (letter K) while refraining from responding to the rare stimuli (letter T). First, participants conduct a baseline block to estimate their mean response time, which later serves as a time limit in the test block. In case participants respond slower than the individual time limit, visual feedback prompts them to respond faster (which usually leads to a higher rate of false alarm, ie, failure of inhibition).

##### Spatial Selective Attention (Visual Search) [101,102]

The visual search task measures visual selective attention. Participants search for a target item presented together with 8 distractor items (a matrix consisting of colored arrows with different orientations). In half of the trials, 1 of 2 predefined targets are present, whereas in the other half, only distractors are presented. Subjects respond to indicate that they have detected a target. The 2 dependent measures that are most commonly studied are reaction time and the ratio of detected targets.

For the measurement on day 1, the EEG is recorded from 64 electrodes positioned according to the extended 10-20 system using a Brain Amp DC amplifier. The data are filtered online at 250 Hz DC, and the sampling rate is set at 1000 Hz. On day 2, the EEG is recorded from 32 active electrodes positioned according to the extended 10-20 system, using a BioSemi system (BioSemi Instrumentation). The sampling rate is 2048 Hz. A common mode sense (CMS) active electrode and a driven right leg (DRL) passive electrode are used. These 2 electrodes form a feedback loop, which drives the average potential of the subject. The reference and ground electrodes are integrated into the CMS und DRL loop. For the follow-up measurements, an extended electrode montage with 64 channels will be used.

#### Structural and Functional MRI

With the start of the second measurement series beginning in late 2021, structural and functional MRI will be included to complement previous neurocognitive measurements. The resting-state MRI data will be recorded to quantify functional network properties and multishell diffusion-weighted imaging as well as multiparametric mapping (qMRI) to estimate the local and network architecture of gray and white matter. In addition, arterial spin labeling will be performed to quantify cerebral blood flow and magnetic resonance spectroscopy to record various parameters of brain metabolism. In the abdomen, the distribution and concentration of visceral and subcutaneous fat as well as the fat and iron concentration of the liver will be measured.

#### Measurement of Biological Parameters

##### Physical Fitness Test, and Cardiovascular and Anthropometric Parameters

Participants’ current physical performance is measured using a bicycle ergometer with a physical work capacity (PWC-130) cycle test. The aim of this test is to predict the absolute power output at a projected heart rate of 130 beats per minute. The relative power output is calculated by the power-to-weight ratio. In addition, the heart rate, electrocardiography (ECG) during rest, as well as the systolic and diastolic blood pressures before and during ergometry are measured. Additionally, the height, weight, waist-to-hip ratio, and BMI are obtained from each participant.

##### Immunological Parameters

We collected 80 ml peripheral venous blood from all participants. Immunological parameters are determined by analyzing the absolute numbers of lymphocytes, quantitative and qualitative changes in the composition of lymphocyte subsets, concentration of cytokines in serum, namely interleukin (IL)-1b, interferon (IFN)-alpha, IFN-gamma, tumor necrosis factor (TNF)-alpha, monocyte chemoattractant protein (MCP-1, IL-6, IL-8, IL-10, IL-12p70, IL-17A, IL-18, IL-23, and IL-33) and functional activities of T cells and natural killer cells [103]. A metric of immune age [35], which was recently developed from a longitudinal cohort using cell subset phenotyping, functional responses of cells to cytokine stimulations and whole blood gene expression data, was adapted to our data by approximation using principal component regression based on the composition of lymphocyte subsets (natural killer and T cells, CD4+, CD8+, and CD8+CD28 T cells). Additionally, peripheral blood mononuclear cells (CD14 positive monocytes) as well as proteins (C-reactive protein), RNA, and DNA are extracted and analyzed using Omics methods.

##### Metabolic Parameters

A urine sample is collected early in the morning before starting the tests, from which creatinine and calcium oxalate are extracted later. Furthermore, ammonia concentration, leucocytes, erythrocytes, hematocrit, monocytes, lymphocytes, cell volume, thrombocytes, triglycerides, cholesterol, high- and low-density lipoprotein cholesterol, glycosylated hemoglobin, glucose, C-reactive protein, and creatinine are measured in venous blood. Later, we plan to measure choline, betaine, and glycerophosphocholine in the blood.

##### Endocrine Parameters

If possible, a hair sample is taken from which hair cortisol concentrations are measured as an index of long-term stress [104].

##### Infections

The presence of a latent *Toxoplasma gondii* infection is explored using specific IgG antibodies. In the follow-up testing, a measure of COVID-19 antibodies will also be obtained.

##### Genetic Parameters (SNPs)

Isolation of genomic DNA of leukocytes is performed according to standard procedures [105]. Several genetic polymorphisms potentially related to the structure and function of the central nervous system are selected. The analyzed SNPs are presented in Table 2.

**Table 2:** Single nucleotide polymorphisms measured in the Dortmund Vital Study (N=528) with the number of observed and expected common homozygotes, heterozygotes, and rare homozygotes in parentheses computed using the Hardy-Weinberg equilibrium calculated on the internet [46]^a^.

Genotype	rs (reference SNP^b^ cluster ID)	Allele observed (n)	Allele expected (n)	χ^2^ (*df*)
**Apo^c^-E2, E3, E4**				
	7412	0=CC (443), 1=CT (83), 2=TT (3)	0=CC (444), 1=CT (81), 2=TT (4)	0.14 (2)
	429358	0=CC (10), 1=CT (127), 2=TT (389)	0=CC (10), 1=CT (126), 2=TT (389)	0.01 (2)
BDNF^d^ Val^66^Met	6265	0=GG (365), 1=GA (148), 2=AA (15)	0=GG (365), 1=GA (148), 2=AA (15)	0.00 (2)
COMT-1^e^	4633	0=CC (128), 1=CT (256), 2=TT (144)	0=CC (124), 1=CT (263), 2=TT (140)	0.45 (2)
COMT-2 Val^158^Met	4680	0=AA (144), 1=AG (256), 2=GG (128)	0=AA (140), 1=AG (263), 2=GG (124)	0.45 (2)
DRD2^f^	6277	0=CC (112), 1=CT (275), 2=TT (141)	0=CC (118), 1=CT (263), 2=TT (146)	1.06 (2)
DRD1^g^-48A/G	4532	0=CC (39), 1=CT (129), 2=TT (93)	0=CC (41), 1=CT (124), 2=TT (95)	0.27 (2)
CHRNA6^h^-1	1072003	0=CC (346), 1=CG (161), 2=GG (21)	0=CC (344), 1=CG (163), 2=GG (19)	0.17 (2)
CHRNA6-3	2304297	0=AA (32), 1=AG (200), 2=GG (296)	0=AA (33), 1=AG (198), 2=GG (297)	0.05 (2)
CHRNB3^i^-1	13280604	0=AA (311), 1=AG (192), 2=GG (25)	0=AA (314), 1=AG (186), 2=GG (28)	0.40 (2)
CHRNB3-2	4950	0=AA (311), 1=AG (191), 2=GG (26)	0=AA (313), 1=AG (187), 2=GG (28)	0.23 (2)
GPCPD1^j^ (EDI3)	6116869	0=GG (198), 1=GT (249), 2=TT (79)	0=GG (198), 1=GT (249), 2=TT (79)	0.00 (2)
GRIN2A^k^	1969060	0=CC (19), 1=CT (165), 2=TT (342)	0=CC (19), 1=CT (164), 2=TT (343)	0.02 (2)
GRIN2A	8057394	0=GG (285), 1=GC (207), 2=CC (36)	0=GG (286), 1=GC (205), 2=CC (37)	0.03 (2)
GRIN2B^l^	890	0=GG (126), 1=TG (254), 2=TT (148)	0=GG (121), 1=TG (263), 2=TT (143)	0.69 (2)
IL^m^-1beta	16944	0=GG (235), 1=GA (221), 2=AA (70)	0=GG (227), 1=GA (237), 2=AA (62)	2.43 (2)
IL-6	1800795	0=CC (85), 1=CG (265), 2=GG (176)	0=CC (90), 1=CG (255), 2=GG (181)	0.78 (2)
IL-12A	568408	0=AA (9), 1=AG (141), 2=GG (178)	0=AA (19), 1=AG (120), 2=GG (188)	*9.53* (2)
TNF-alpha^n^	1800629	0=AA (15), 1=AG (141), 2=GG (372)	0=AA (14), 1=AG (143), 2=GG (370)	0.13 (2)

^a^The chi-square test indicates the conformity between the expected and observed distribution. Significant deviances from the HWE are italicized.

^b^SNP: single nucleotide polymorphism.

^c^APO: apolipoprotein.

^d^BDNF: brain-derived neurotrophic factor.

^e^COMT: catechol-O-methyltransferase.

^f^DRD2: dopamine receptor D2.

^g^DRD1: dopamine receptor D1.

^h^CHRNA6: cholinergic receptor nicotinic alpha 6.

^i^CHRNB3: cholinergic receptor nicotinic beta 3.

^j^GPCPD1: glycerophosphocholine phosphodiesterase.

^k^GRIN2A: glutamate ionotropic receptor NMDA type subunit 2A.

^l^GRIN2B: glutamate ionotropic receptor NMDA type subunit 2B.

^m^IL: interleukin.

^n^TNF-alpha: tumor necrosis factor-alpha.

In future analyses, we intend to evaluate polygenic scores (PGS) because based on the findings of several recent large-scale genome-wide association studies, it became increasingly clear that thousands of alleles across the genome (polygenic architecture) contribute to interindividual variations in cognitive performance with small effect sizes [19]. DNA genotyping will be carried out using the Illumina Infinium Global Screening Array 3.0 with major depressive disorder and Psych content, and genome-wide PGS (weighted sums of each participant's trait-associated alleles across all SNPs) will be created using publicly available summary statistics for cognitive performance and other available behavioral phenotypes of interest.

### Research Data Management

#### Types of Data

The types of data generated are qualitative and quantitative questionnaire data, sensory and psychometric scores, behavioral data from psychological experiments, EEG data, MRI data (starting with the second measurement wave), concentrations of immunological parameters, endocrinological data, genetic polymorphisms and PGS, ECG and cardiovascular data, anthropometric data, and the concentrations of metabolites.

Measures taken for quality assurance and quality management comprise continuous control of raw data, sample data analysis, data validation through split-half, test-retest reliability between measures, and data validation by statistical analysis.

The used formats of the generated EEG data are MATLAB (*.m), BrainVision Core Data Format (each recording consisting of a *.vhdr, *.vmrk, *.eeg file triplet) and BioSemi EEG data (*.bdf). SPSS (*.sav, *.spv) and R-data are used for statistical analyses. Integration of all data types takes place in a Structured Query Language (SQL) database. If appropriate, raw data (eg, immunological data, SNPs, questionnaires, and neuropsychological tests) are included in the database. Some types of data, namely preprocessed EEG data, are included in the database after averaging for each person, task, and condition separately. Useful variables and total scores are extracted from the large number of questionnaires and neuropsychological tests. The EEG and MRI raw data will be later converted into the Brain Imaging Data Structure (BIDS) format [106,107]. The aim of BIDS is to create standards allowing researchers to readily organize and share study data within and between laboratories.

#### Storage, Selection, and Retention Period

All data are stored on IfADo servers as working copies, as proof of good scientific practice, for reuse, and for legal and contractual requirements. Research data are stored and analyzed without reference to the personal data of the subjects. Personal subject information (such as name and contact data required for re-invitation) are physically separated from the research data (pseudonymization). Only a few selected persons have access to the subject’s identities and the subject IDs that are stored in written form in a secure location. The anonymized research data are stored in server rooms at different locations throughout the institute. The amount of data is approximately 1 TB per year excluding MRI data, which is estimated to be approximately 2 TB per year. Technologies used for data storage include an NAS (Network Attached Storage) server with the RAID5 (redundant array of independent disks) configuration. Additional backup copies are regularly made and checked. Paper and pencil tests and questionnaires are stored in a central archive of the institute. Isolated peripheral blood mononuclear cells, serum, and urine probes are frozen for future use.

#### Data Access and Use

To access the data, scientists from IfADo, as well as external cooperation partners who plan to analyze data from the Dortmund Vital Study fill in a proposal form that includes a short description of the project and the respective hypotheses, responsible persons, cooperation partners, data usage, and analysis strategy. The requested research data will be made available in an anonymized form after consultation with the scientists responsible for the data. Responsible persons include project managers and coordinators of the Dortmund Vital Study in consultation with the IfADo Research Data Management Unit.

After primary analyses and publication of the main results, the data and the scripts used for data analyses will be made available in repositories for secondary analyses. The transfer agreement will be prepared by the coordinators of the Dortmund Vital Study in consultation with the IfADo Research Data Management Unit. Data access will be restricted and will require a structured project proposal. Access to the SQL database will be password protected. Interoperability will be guaranteed by metadata included in the SQL database and common data formats (eg, BIDS) to enhance exchange, management, and documentation. Data identifiers (digital object identifiers) will be assigned at least until the end of the projects.

#### Organization, Management, and Policies

Central organizational support for data management is provided by the Research Data Management Unit at IfADo.

The Dortmund Vital Study is funded by the institute’s budget. Thus, the study design, collection, management, analysis, interpretation of data, writing of the report, and the decision to submit the report for publication is not influenced by or biased toward any sponsor.

Permissions for the tests, questionnaires, and software used that are subject to copyright were obtained from the corresponding publishers. If no license was required, permission was granted by the authors of the corresponding questionnaire or test.

### Data Analysis

Detailed descriptions of the specific data analysis methods depending on the type of measure will be provided in subsequent publications.

Generally, for all quantitative variables, descriptive statistics, frequencies, and distributions will be calculated for all participants. Summary statistics including the mean, SD, minimum values, and maximum values will be provided for quality assurance. Qualitative variables will be categorized before performing any statistical analysis. Immunological, biochemical, medical, optometric, and audiometric data as well as coding for genetic polymorphisms are integrated in the SQL database. Data from psychometric tests in the SQL database will be verified by using an automated MATLAB script for plausibility and outliers. EEG and behavioral data from experimental tasks will be analyzed with EEGLAB [108] using scripts. The main parameters, such as individual amplitudes or latencies of transient components, time-frequency parameters, or behavioral data, such as reaction times, error rates, and SDs, will be automatically written in the SQL database.

Depending on the research topics and questions, different methods will be used for data analysis. For the cross-sectional analyses, mixed ANOVA, Analyses of Covariance (ANCOVA), 1-way ANOVA, or *t* tests will be used. Furthermore, the Pearson correlation coefficient or appropriate regression models will be used to identify predictors of the variables of interest. Moreover, the structural equation modeling approach and novel decoding techniques will be employed when appropriate.

For the longitudinal data, mixed model ANOVA including within-subject factors, such as Time Point of Measure, and between-subject factors, such as Age Group, will be employed. Interactions will be analyzed using simple ANOVA or *t* tests. Additionally, covariance analysis (ANCOVA) will be conducted to control for potential confounding factors like gender and education. It is not intended to impute missing data for ANOVA or ANCOVA. In case of a few or moderate number of missing data, the analyses will be conducted with the complete cases. In case of a larger number of missing data over the long period, advanced statistical approaches like the Generalized Estimating Equation (GEE) [109] or Mixed Effect Regression (MER) [110] will be used.

### Ethics Approval

The first run of the Dortmund Vital Study (2016-2021) was conducted with approval from the local Ethics Committee of the IfADo in 2015. Follow-up testing was approved in July 2021 by the Ethics Committee of the IfADo (approval number: A93-3).

## Results

The baseline testing was conducted between 2016 and 2021. As of August 2021, 593 participants were tested. In October 2021, the first follow-up measure was started. Some initial results from the cross-sectional part of the study were already published [83, 111-116]. Longitudinal data will be analyzed, and the first publications are estimated for 2025.

## Discussion

### Study Overview

The Dortmund Vital Study is a multidisciplinary long-term project, combining a cross-sectional and longitudinal study design. The goal is to investigate the influence of a wide range of biological and environmental factors on cognitive functions and their neurophysiological correlates as well as on the immune system and other physiological functions and structures. The combination of well-elaborated experimental paradigms reflecting basic cognitive functions, modern EEG methodology, MRI scans, lifestyle data, and the analysis of biochemical parameters will facilitate the development and evaluation of specific hypotheses on the mechanisms of healthy and pathological aging from different perspectives and disciplines. Work-related human factors (eg, type of work), work ability, and the role of work conditions (eg, stress or job satisfaction) will be of particular interest. Moreover, the Dortmund Vital Study has the potential to shed tremendous light on the complex interactions between immunological, genetic, metabolic, and brain-related parameters in healthy adults. Specifically, parameters obtained by MRI and EEG-related measures can be evaluated as a function of the PGS, metabolic products, concentration of immune cells, immune age, and infections such as Toxoplasmosis or COVID-19 that are largely unexplored. The same is true for environmental and lifestyle factors that influence brain activity and behavior. As human behavior and cognition reflects a combination of these factors, the Dortmund Vital Study has the potential to elucidate several important findings. Crucial points are the developmental aspect and the progress or decline of several functions over the lifespan. This design enables analysis of changes of inter- and intraindividual variability with increasing age and lead to conclusions, such as which factors in the past have the largest impact on the present measures or pathological outcomes like burnout, depression, mild cognitive impairment, or dementia. In conclusion, this multidomain and interdisciplinary study will help elucidate underlying mechanisms of healthy and pathological aging.

### Limitations and Future Challenges

A challenging problem in longitudinal study designs is the dropout rate, especially during long test periods like in this study. The dropout rate can be affected by unpredictable events like the present COVID-19 pandemic. A reduced number of participants during follow-up produces missing values, shrinks the entire sample size, reduces the statistical power, and may lead to a small sample size at the end of the study relative to the number of analyzed variables.

Thus, it is important to analyze the reasons for dropouts and whether the participants are missing at random or not at random (ie, due to unsystematic or systematic reasons), which may differently affect the results. For example, dropouts due to lack of interest in further participation in the study or change of residence can be treated as random. In contrast, dropouts due to illness or death could reflect a systematic reason for missing data, given that elderly participants entering the study have a much higher risk of illness or death in the long period spanning 20 years. This may lead to a disproportionate dropout in this age group compared to the younger groups. Moreover, a pandemic like COVID-19 reflects a systematic source of dropouts.

Missing data have important consequences for data analysis. Repeated measures ANOVA or MANOVA requires fully complete data sets for each repeated measure. That is, without data imputation, only a reduced (but complete) data set can be used for analysis. There are several methods to counteract this using statistical approaches. For example, the missing data can be replaced by the last measured data or by a simple or conditional mean of the variable resulting from a predicted value. However, data imputation is generally not recommended, and instead, other methods should be used to deal with missing data sets [117].

For the present design, a GEE [109] model will be used, which is robust to misspecifications of the repeated measures’ correlation structure, time irregularities, and does not require excluding participants with incomplete data sets. An alternative method would be MER [110], in which random effects can serve to describe each participant’s trend over time. MER assesses longitudinal changes of several outcomes and is even more robust to missing data than the GEE. Both models allow time-invariant predictors (eg, biological gender, genotype) and time-varying predictors (eg, age, metabolic, immunological, or cognitive parameters), and handle irregularly timed and missing data without the need for imputation [118]. An advantage of these procedures is that both can model time-varying predictions useful for understanding changes in cognitive aging.

Nevertheless, the relatively large number of participants in this study that primarily aims to analyze cognitive changes using neuropsychology, EEG, and MRI is unique and even taking dropouts into account allows answering important scientific questions. For example, when interested in age-related changes in an electrophysiological parameter, a group of approximately 40 participants would still be sufficient to track the changes across the span of 20 years. Generally, we expect the dropout rate to remain constant and to be able to analyze the data with a sufficiently large sample at least for the first follow-up measures 5 and 10 years after the beginning of the study.

## Abbreviations

ANCOVA: Analyses of Covariance
ANOVA: analyses of variance
AX-CPT: AX-continuous performance task
BDNF: brain-derived neurotrophic factor
CMS: Common Mode Sense
COMT: catechol-O-methyltransferase
DRL: driven right leg
ECG: electrocardiography
EEG: electroencephalography
HAWIE-R: Hamburg-Wechsler-Intelligenztest für Erwachsene, Revision
HWE: Hardy-Weinberg equilibrium
GEE: Generalized Estimating Equation
IFN: interferon
IL: interleukin
LPS: Leistungs-Prüf-System
MER: Mixed Effect Regression
MMSE: Mini Mental State Examination
MRI: magnetic resonance imaging
MWT-B: Multiple-Choice Vocabulary Test
NAS: Network Attached Storage
PGS: polygenic scores
RAVLT: Rey Auditory Verbal Learning Test
SNP: single nucleotide polymorphism
TNF: tumor necrosis factor
VLMT: Verbal Learning and Memory Test
WAI: Work Ability Index
WAIS-III: Wechsler Adult Intelligence Scale
WM: working memory
